# KMT2A/C mutations function as a potential predictive biomarker for immunotherapy in solid tumors

**DOI:** 10.1186/s40364-020-00241-0

**Published:** 2020-12-09

**Authors:** Rui Zhang, Hao-Xiang Wu, Ming Xu, Xiaoyan Xie

**Affiliations:** 1 Department of Medical Ultrasound, Division of Interventional Ultrasound, The First Affiliated Hospital, Sun Yat-sen University, No. 58 Zhongshan 2nd Road, Guangzhou, 510080 China; 2 Department of Clinical Research, State Key Laboratory of Oncology in South China, Collaborative Innovation Center for Cancer Medicine, Sun Yat-sen University Cancer Center, 510060 Guangzhou, China

**Keywords:** Biomarker, Immune checkpoint inhibitors, KMT2A/C, Pan-cancer analysis

## Abstract

Epigenetic factors play important roles in tumor immunology. Histone-lysine N-methyltransferase 2 (KMT2) family genes exert histone H3 methylation, but its role in immunotherapy remains unclear. Our study is the first to investigate the correlation between KMT2 gene mutations and the clinical benefit of immune checkpoint inhibitors (ICI) treatment. We firstly collected a primary ICI-treated cohort (*n* = 546) and found that patients with KMT2A/C mutations yielded better prognosis in terms of progression-free survival (PFS, Hazard ratio [HR] = 0.66, *P* = 0.002), objective response rate (ORR, 40.9% vs 20.3%, *P* < 0.001), durable clinical benefit (DCB, 48.3% vs 29.8%, *P* = 0.001) and overall survival (OS, HR = 0.70, *P* = 0.033). Furthermore, we validated the predictive potential of KMT2A/C mutations in an expanded ICI-treated cohort (*n* = 1395). KMT2A/C-mutant patients achieved better OS compared with KMT2A/C-wildtype patients (HR = 0.68, *P* = 0.003); and the survival advantages appeared in the majority of cancer subtypes. Our study suggests that KMT2A/C mutations function as a novel and potential predictive biomarker for ICI treatment in multiple solid tumors and the underlying mechanism is worth investigating.

**To the Editor,**

Recent years have witnessed the great success of immune checkpoint inhibitors (ICIs) in treating multiple advanced tumors [1]. However, clinical response of ICIs varies and identification of predictive biomarkers is still in urgent demand.

Growing evidence suggests that epigenetic factors play important roles in immuno-oncology [2, 3]. For example, the mutation in DNA demethylase TET1 predicted higher response rate in ICI-treated patients [4], and DNA methyltransferase inhibitors (DNMTi) and histone deacetylase inhibitors (HDACi) showed promising potentials to augment the efficacy of ICIs [5, 6].

The KMT2 family genes, one of the important epigenetic regulator genes, were initially recognized in mixed-lineage leukemia (MLL) caused by the rearrangement of KMT2A on chromosome 11q23 [7, 8]. Recent exome-sequencing studies revealed that KMT2 genes were among the most frequently mutated genes in various types of human cancers [9]. The KMT2 proteins, namely KMT2A, KMT2B, KMT2C and KMT2D, function as methylating histone H3 on lysine 4 (H3K4) to promote genome accessibility and transcription, but its role in immunotherapy remains unclear. Herein, we investigated the correlation between KMT2 gene mutations and clinical benefit of ICI treatment in the human pan-cancer setting, which was the first time to the best of our knowledge.

To address this issue, we collected a primary ICI-treated cohort (*n* = 546) with annotated response and mutational data as well as survival data from seven published studies (see Methods), composing of bladder cancer, esophagogastric cancer, head and neck cancer, hepatocellular carcinoma, melanoma and non-small-cell lung cancer (baseline characteristics were shown in Table S1). We found that both KMT2A (*P* = 0.007) and KMT2C (*P* = 0.041) mutations significantly correlated with better progression-free survival (PFS) in patients receiving ICI treatment, while KMT2B (*P* = 0.964) and KMT2D (*P* = 0.200) did not. Then we combined KMT2A and KMT2C mutation as KMT2A/C mutations, and found that patients harboring KMT2A/C mutations correlated with longer PFS most significantly (Fig. 1a, P = 0.002). Moreover, the Bonferroni-corrected *p* value of the association between KMT2A/C-mutant and PFS was 0.010, indicating that KMT2A/C-mutant was robustly associated with improved PFS in patients treated with ICIs.

**Fig. 1:**
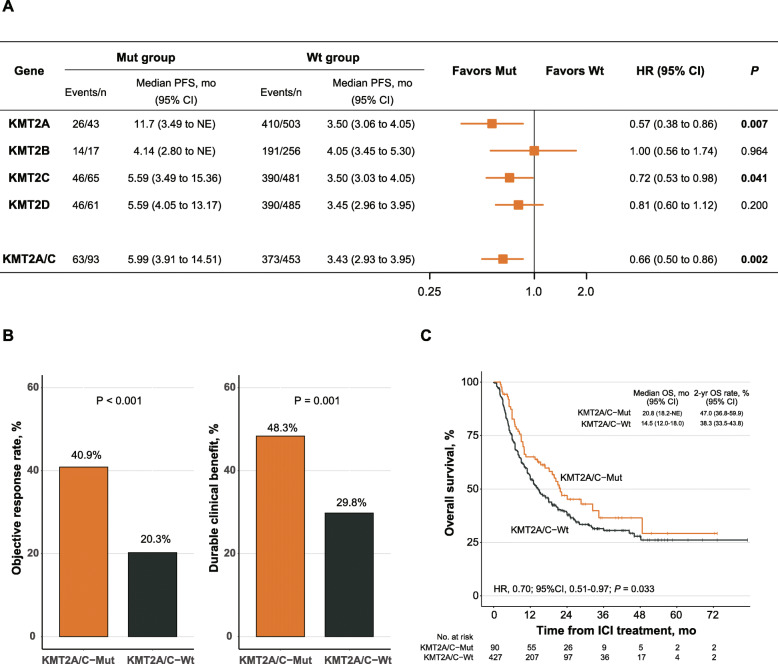
KMT2A/C mutations predicted improved clinical outcomes in patients receiving ICI treatment. **a** The primary ICI-treated cohort (*n* = 546) was divided into the mutant (Mut) group and the wildtype (Wt) group according to the mutation status of individual KMT2 genes and KMT2A/C genes, then the difference of progression-free survival (PFS) between the two groups was compared. **b** Histogram demonstrating the proportions of patients achieving objective response (ORR, left panel) and durable clinical benefit (DCB, right panel) in the KMT2A/C-Mut and the KMT2A/C-Wt groups. **c** Kaplan-Meier curves of overall survival (OS) according to KMT2A/C mutation status in the ICI-treated cohort (*n* = 517 due to the lacking of OS information of 29 patients). Median OS and 2-year OS were also depicted

We subsequently analyzed the association between KMT2A/C mutations and objective response rate (ORR), durable clinical benefit (DCB) and overall survival (OS) in patients receiving ICI treatment. Compared with KMT2A/C-wildtype patients, KMT2A/C-mutant patients achieved significantly higher ORR (40.9% vs. 20.3%, *P* < 0.001; Fig. 1b, left panel), better DCB (48.3% vs. 29.8%, *P* = 0.001; Fig. 1b, right panel) and improved OS (HR = 0.70 [95% confidence interval (CI), 0.51–0.97], *P* = 0.033; Fig. 1c). Thus, our findings suggest that KMT2A/C mutations may function as a potential predictive biomarker for ICI treatment.

To further validate the predictive value of KMT2A/C mutations, we employed an expanded ICI-treated cohort (*n* = 1395) from Memorial Sloan Kettering Cancer Center on the basis of Samstein and his colleagues’ work (see Methods). As shown in Fig. 2a, KMT2A/C-mutant patients achieved significant improved OS (median OS, 41 months [95% CI, 34-NE]) compared with KMT2A/C-wildtype patients (median OS, 17 months [95% CI, 15–21]). In order to investigate whether the improvement of OS in KMT2A/C-mutant patients alters with cancer category, we analyzed OS between KMT2A/C-mutant and KMT2A/C-wildtype in different cancer types. And the favorable clinical outcomes for KMT2A/C-mutant were yielded in the majority of the examined cancer types, the overall HR was 0.68 [95% CI, 0.53–0.88] (*P* = 0.003, Fig. 2b), indicating a 32% lower risk of death. In addition, no survival difference was observed between KMT2A/C-mutant patients and KMT2A/C-wildtype patients in a non-ICI-treated cohort (Figure S1, *n* = 2252, HR = 0.90 [95% CI, 0.71–1.14], *P* = 0.396), confirming that the improvement of OS after ICI treatment in KMT2A/C-mutant patients compared with that in KMT2A/C-wildtype patients was indeed transformed from its higher response rate, not contributed by its general prognostic impact.

**Fig. 2:**
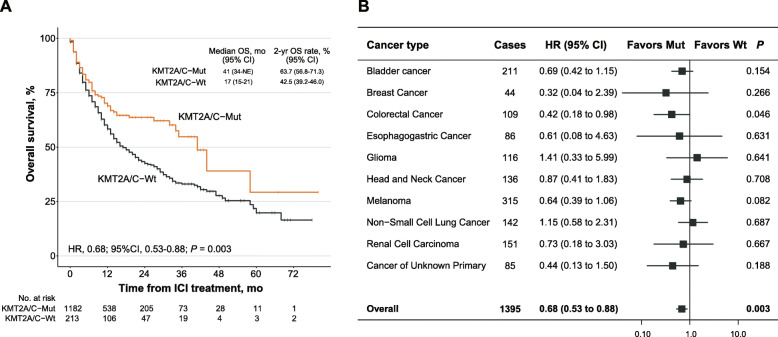
Validation of the predictive function of KMT2A/C mutations in the expanded ICI-treated cohort. **a**. Kaplan-Meier curves comparing the overall survival (OS) between the KMT2A/C-Mut group and the KMT2A/C-Wt group in the expanded ICI-treated cohort (*n* = 1395). mo, months; yr, years. Median OS and 2-year OS were also depicted. **b**. Forest plot depicting the OS benefit of KMT2A/C-Mut patients compared with KMT2A/C-Wt patients by cancer category. Bars represent the 95% CI

The average occurrence frequency of KMT2A/C-mutant was 17.0% and 15.3% in the primary ICI-treated cohort and the expanded ICI-treated cohort, respectively. In addition, we employed the TCGA dataset to investigate the mutational frequency of KMT2A/C in different cancer types. And the results showed that (Figure S2), the mutational frequency of KMT2A/C ranged from 0% (uveal melanoma) to 30.9% (skin cutaneous melanoma) among 33 cancer types (average, 12.2%), which would help in identifying a substantial proportion of cancer patients who would benefit for ICI treatment.

KMT2 genes encoding histone-lysine N-methyltransferase 2 proteins act as writer of histone methylation at important regulatory regions in the genome and thereby impart crucial functions through modulating chromatin structures and DNA accessibility. It’s reported that the majority of KMT2A/C mutations can cause loss of function in the proteins, which would damage its writer function and lead to changes in the transcriptional regulation [10]. Although there is now no direct evidence between KMT2A/C mutations and tumor immunity, we found that KMT2A/C-mutant tumors tend to have higher tumor mutational burden (TMB) and better clinical outcomes in patients treated with ICI compared with KMT2A/C-wildtype tumors (Figure S3), which suggested that KMT2A/C-mutant may sensitize tumors to ICI treatment at least partially by increasing tumor immunogenicity. As TMB-high has already been established as solid predictive biomarker for ICI treatment, we further evaluated the predictive function of KMT2A/C-mutant in TMB-low patients and found that KMT2A/C-mutant could still be predictive of better ORR (32.6% vs 17.9%, *P* = 0.029) in the TMB-low subgroup (Figure S4), suggesting the further predictive value of KMT2A/C-mutant beyond TMB and that other possible mechanisms besides higher immunogenicity could also contribute to the sensitivity of KMT2A/C-mutant to ICI treatment, which warranted further investigation.

This study also has several limitations. Firstly, no available information of PD-L1 expression could be included in our study for comparison, though PD-L1 expression seems to be an “imprecise” predictor of response to ICI treatment [11, 12]. Secondly, although more than 10 cancer types with ICI data were involved in our analysis, more data are needed concerning the potential predictive function of KMT2A/C-mutant in other cancer types. Our study should be considered as generators of hypotheses, and the best way to confirm this hypothesis is a biomaker-driven and tumor histology-agnostic clinical trial incorporating KMT2A/C-mutant as biomarker in patients receiving ICI treatment.

In conclusion, our study is the first to reveal the correlation between KMT2A/C mutations and ICI treatment efficacy, which suggest that KMT2A/C mutations can function as a novel and potential biomarker in multiple solid tumors, and adds great value in the identification of patients who might benefit from ICI treatment and may guide the clinical decision-making on the use of ICI. Further studies on the underlying molecular mechanism that KMT2A/C mutations sensitize patients to ICI treatment are still warranted.

## Supplementary Information

**Additional file 1. Methods**.

**Additional file 2: Table S1.** Patient characteristics between KMT2A/C-Wt and KMT2A/C-Mut subgroups of the primary ICI-treated cohort. (*n* = 546).

**Additional file 3: Figure S1.** Kaplan-Meier curves comparing the overall survival (OS) between the KMT2A/C-Mut group and the KMT2A/C-Wt group in the non-ICI-treated cohort (*n* = 2252).

**Additional file 4: Figure S2.** The mutational frequency of KMT2A/C across 33 cancer types in the TCGA cohort.

**Additional file 5: Figure S3.** Boxplot comparing the tumor mutational burden (TMB) between KMT2A/C-Mut tumors and the KMT2A/C-Wt tumors in the primary ICI-treated cohort.

**Additional file 6: Figure S4.** Barplot comparing the objective response rate (ORR) between KMT2A/C-Mut patients and the KMT2A/C-Wt patients in the TMB-low subgroup of the primary ICI-treated cohort.

## Data Availability

All of the data used in this study were publicly available in cBioPortal and the analysis and interpretation of data was described in the Methods section (See supplementary files).

## Abbreviations

CI: Confidence interval
DCB: Durable clinical benefit
DNMTi: DNA methyltransferase inhibitor
HDACi: Histone deacetylase inhibitors
HR: Hazard ratio
ICI: Immune checkpoint inhibitors
MLL: Mixed-lineage leukemia
H3K4: Histone H3 on lysine 4
KMT2: Histone-lysine N-methyltransferase 2
Mut: Mutant
NE: Not evaluable
ORR: Objective response rate
OS: Overall survival
PFS: Progression-free survival
TMB: Tumor mutational burden
Wt: Wildtype
