# Supplementary material for: KMT2A/C mutations function as a potential predictive biomarker for immunotherapy in solid tumors
Source: Biomark Res. 2020 Dec 9;8:71. doi: 10.1186/s40364-020-00241-0 (PMC7724704; doi:10.1186/s40364-020-00241-0)
Supplement: Supplementary file 1 — Additional file 1. Methods. [file 40364_2020_241_MOESM1_ESM.docx]

# Methods

**Collection of clinical cohorts**

Seven published cohorts[[1-7](#_ENREF_1)] with mutational data of KMT2 genes, corresponding response and survival data of patients receiving immune checkpoint inhibitors (ICI) treatment were merged together to form the primary ICI-treated cohort*.* The main inclusion criteria are as follows, (1) patients receiving ICI treatment (anti-CTLA-4, anti-PD-[L]1 or their combination), (2) patients with annotated response and survival data after immunotherapy, (3) patients with information on the mutational status of KMT2 genes. Based on the above inclusion criteria, 27 patients with hepatocellular carcinoma, 40 patients with esophagogastric cancer, 240 patients with non-small cell lung cancer from the first three cohorts were included; besides, 249 patients with mixed cancer types from the latter four cohorts were further included, in which patients without qualified mutational data or response data were already filtered out by Miao D. et al.[[7](#_ENREF_7)]. In total, 556 patients were pre-included in the primary ICI-treated cohort, in which we further excluded patients receiving concurrent therapy besides ICIs (n = 3) and cancer type with patients less than 10 (n = 7). In the end, 546 eligible patients were included in the primary ICI-treated cohort. The genomic sequencing methods used in these studies included MSK-IMPACT panel and whole-exome sequencing (WES). And the total number of nonsynonymous somatic mutations were normalized by the corresponding exonic coverage to derive tumor mutational burden (TMB) as previously described[[8](#_ENREF_8)]. The cutoff of the top 20% within each histology was used to divided patients into TMB-high and TMB-low groups as proposed by Samstein et al.[[9](#_ENREF_9)].

For validation, an expanded ICI-treated cohort (n=1395) and the a non-ICI-treated cohort (n=2252) were obtained from Samstein et al.[[9](#_ENREF_9)] and filtered and consolidated as described in our previous study[[8](#_ENREF_8)].

**KMT2 genes mutations**

Patients with nonsynonymous somatic mutations in the coding region of KMT2 genes, including missense, nonsense, nonstop, splice site mutations and inframe and frameshift insertion and deletion, were defined as KMT2-mutant (KMT2-Mut), and patients without as KMT2-wildtype (KMT2-Wt)[[10](#_ENREF_10)]. The TCGA somatic mutational data from Ellrott et al.[[11](#_ENREF_11)] was employed to evaluate the mutational frequency of KMT2A/C across multiple cancer types.

**Statistical analysis**

We analyzed the association between KMT2 gene status and the efficacy of ICI treatment, including objective response rate (ORR), durable clinical benefit (DCB), progression-free survival (PFS) and overall survival (OS)[[8](#_ENREF_8)]. The association between KMT2 gene status and ORR or DCB were examined using fisher’s exact test. The progression-free and overall survival probability of Mut and Wt patients were analyzed by Kaplan-Meier method, log-rank test, and Cox proportional hazards regression analysis. Besides, available confounding factors were collected and adjusted to derived the adjusted hazard ratio (HR) and P value, including (1) age, sex, and cancer type in the primary ICI-treated cohort; (2) age, sex, and cancer type in the expanded ICI-treated cohort; (3) sex and cancer type in the non-ICI-treated cohort. Two-sided P value < 0.05 was considered statistically significant. All the statistical analyses were conducted with R v. 3.6.1 (<http://www.r-project.org>).

# Supplementary references

1. Harding J, Nandakumar S, Armenia J, Khalil D, Albano M, Ly M, et al. Prospective Genotyping of Hepatocellular Carcinoma: Clinical Implications of Next-Generation Sequencing for Matching Patients to Targeted and Immune Therapies. Clinical cancer research : an official journal of the American Association for Cancer Research 2019;25(7):2116-26.

2. Janjigian Y, Sanchez-Vega F, Jonsson P, Chatila W, Hechtman J, Ku G, et al. Genetic Predictors of Response to Systemic Therapy in Esophagogastric Cancer. Cancer discovery 2018;8(1):49-58.

3. Rizvi H, Sanchez-Vega F, La K, Chatila W, Jonsson P, Halpenny D, et al. Molecular Determinants of Response to Anti-Programmed Cell Death (PD)-1 and Anti-Programmed Death-Ligand 1 (PD-L1) Blockade in Patients With Non-Small-Cell Lung Cancer Profiled With Targeted Next-Generation Sequencing. Journal of clinical oncology : official journal of the American Society of Clinical Oncology 2018;36(7):633-41.

4. Rizvi N, Hellmann M, Snyder A, Kvistborg P, Makarov V, Havel J, et al. Cancer immunology. Mutational landscape determines sensitivity to PD-1 blockade in non-small cell lung cancer. Science (New York, N.Y.) 2015;348(6230):124-8.

5. Snyder A, Makarov V, Merghoub T, Yuan J, Zaretsky J, Desrichard A, et al. Genetic basis for clinical response to CTLA-4 blockade in melanoma. The New England journal of medicine 2014;371(23):2189-99.

6. Van Allen E, Miao D, Schilling B, Shukla S, Blank C, Zimmer L, et al. Genomic correlates of response to CTLA-4 blockade in metastatic melanoma. Science (New York, N.Y.) 2015;350(6257):207-11.

7. Miao D, Margolis C, Vokes N, Liu D, Taylor-Weiner A, Wankowicz S, et al. Genomic correlates of response to immune checkpoint blockade in microsatellite-stable solid tumors. Nature genetics 2018;50(9):1271-81.

8. Wu H, Chen Y, Wang Z, Zhao Q, He M, Wang Y, et al. Alteration in TET1 as potential biomarker for immune checkpoint blockade in multiple cancers. Journal for immunotherapy of cancer 2019;7(1):264.

9. Samstein R, Lee C, Shoushtari A, Hellmann M, Shen R, Janjigian Y, et al. Tumor mutational load predicts survival after immunotherapy across multiple cancer types. Nature genetics 2019;51(2):202-06.

10. Riaz N, Havel J, Kendall S, Makarov V, Walsh L, Desrichard A, et al. Recurrent SERPINB3 and SERPINB4 mutations in patients who respond to anti-CTLA4 immunotherapy. Nature genetics 2016;48(11):1327-29.

11. Ellrott K, Bailey MH, Saksena G, Covington KR, Kandoth C, Stewart C, et al. Scalable Open Science Approach for Mutation Calling of Tumor Exomes Using Multiple Genomic Pipelines. Cell Syst 2018;6(3):271-81.e7.
