# Supplementary material for: KMT2A/C mutations function as a potential predictive biomarker for immunotherapy in solid tumors
Source: Biomark Res. 2020 Dec 9;8:71. doi: 10.1186/s40364-020-00241-0 (PMC7724704; doi:10.1186/s40364-020-00241-0)
Supplement: Supplementary file 2 — Additional file 2: Table S1. Patient characteristics between KMT2A/C-Wt and KMT2A/C-Mut subgroups of the primary ICI-treated cohort. (n = 546). [file 40364_2020_241_MOESM2_ESM.docx]

**Table S1. Patient characteristics between KMT2A/C-Wt and KMT2A/C-Mut subgroups of the ICI-treated cohort (n = 546).**

| **Characteristics** | **KMT2A/C status (No. [%]^#^)** | | ***P* value^*^** |
| --- | --- | --- | --- |
|  | **Wildtype** | **Mutant** |  |
| **No. of patients** | 453 (83.0) | 93 (17.0) | - |
| **Age (yrs)** |  |  | 0.682 |
| <=30 | 8 (80.0) | 2 (20.0) |  |
| 31-50 | 66 (85.7) | 11 (14.3) |  |
| 51-60 | 87 (81.3) | 20 (18.7) |  |
| 61-70 | 119 (81.5) | 27 (18.5) |  |
| >70 | 109 (81.3) | 25 (18.7) |  |
| Unknown | 64 (88.9) | 8 (11.1) |  |
| **Sex** |  |  | 0.646 |
| Male | 260 (82.3) | 56 (17.7) |  |
| Female | 193 (83.9) | 37 (16.1) |  |
| **Cancer type** |  |  | 0.005 |
| Bladder cancer | 18 (66.7) | 9 (33.3) |  |
| Esophagogastric cancer | 33 (82.5) | 7 (17.5) |  |
| Head and neck cancer | 10 (100.0) | 0 (0.0) |  |
| Hepatocellular carcinoma | 25 (92.6) | 2 (7.4) |  |
| Melanoma | 112 (75.7) | 36 (24.3) |  |
| Non-small-cell lung cancer | 255 (86.7) | 39 (13.3) |  |
| **Best overall response** |  |  | 0.001 |
| CR | 19 (70.4) | 8 (29.6) |  |
| PR | 72 (70.6) | 30 (29.4) |  |
| SD | 128 (87.7) | 18 (12.3) |  |
| PD | 230 (86.1) | 37 (13.9) |  |
| NE | 4 (100.0) | 0 (0.0) |  |
| **Durable clinical benefit** |  |  | 0.003 |
| DCB | 128 (74.9) | 43 (25.1) |  |
| NDB | 302 (86.8) | 46 (13.2) |  |
| NE | 23 (85.2) | 4 (14.8) |  |

^#^ Indicated percentage of patients with KMT2A/C-Mut amongst total patients within a given category (i.e. a specific cancer type, or a specific sex).

* Fisher’s exact test for categorical variables. *P* value < 0.05 denoted statistically significant differences in a given patient characteristics between KMT2A/C-Mut and KMT2A/C-Wt subgroups.

Abbreviations: CR, complete response; DCB, durable clinical benefit; NDB, no durable benefit; NE, not evaluable; PD, progressive disease; PR, partial response; SD, stable disease.
