# Supplementary figures and images for: KMT2A/C mutations function as a potential predictive biomarker for immunotherapy in solid tumors
Source: Biomark Res. 2020 Dec 9;8:71. doi: 10.1186/s40364-020-00241-0 (PMC7724704; doi:10.1186/s40364-020-00241-0)

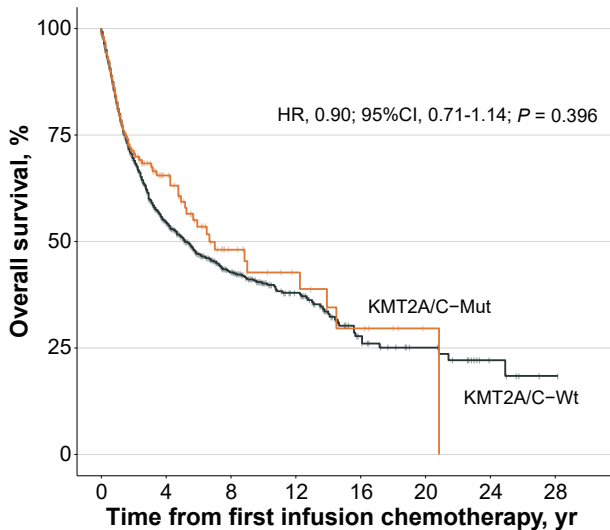

|             |      |     |     |    |    |    |   |   |  |
|-------------|------|-----|-----|----|----|----|---|---|--|
| No. at risk |      |     |     |    |    |    |   |   |  |
| KMT2A/C-Mut | 2049 | 452 | 174 | 94 | 32 | 18 | 6 | 1 |  |
| KMT2A/C-Wt  | 203  | 58  | 21  | 11 | 6  | 1  | 0 | 0 |  |

Supplement: Supplementary file 3 — Additional file 3: Figure S1. Kaplan-Meier curves comparing the overall survival (OS) between the KMT2A/C-Mut group and the KMT2A/C-Wt group in the non-ICI-treated cohort (n = 2252). [file 40364_2020_241_MOESM3_ESM.pdf]

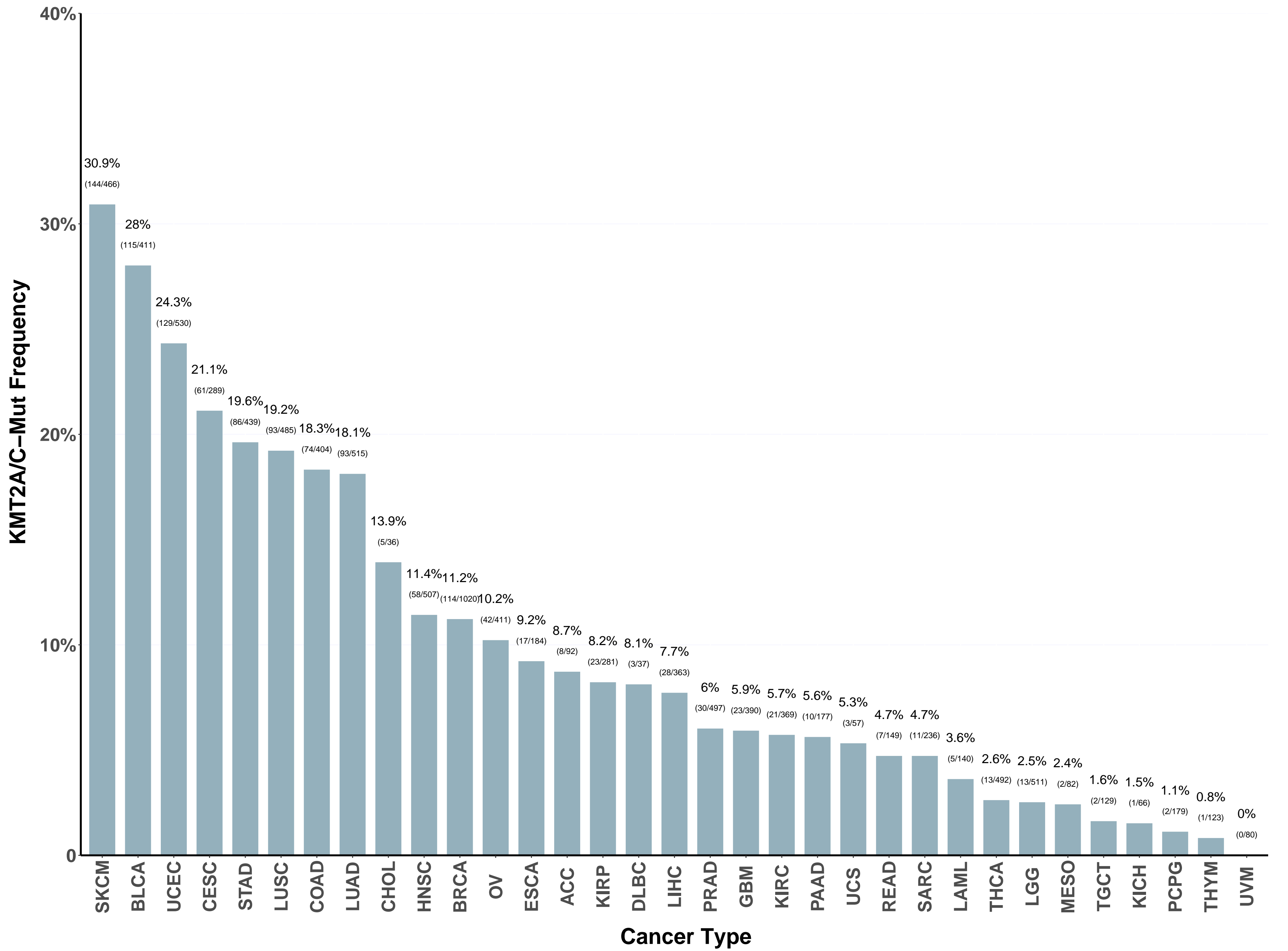

Supplement: Supplementary file 4 — Additional file 4: Figure S2. The mutational frequency of KMT2A/C across 33 cancer types in the TCGA cohort. [file 40364_2020_241_MOESM4_ESM.pdf]

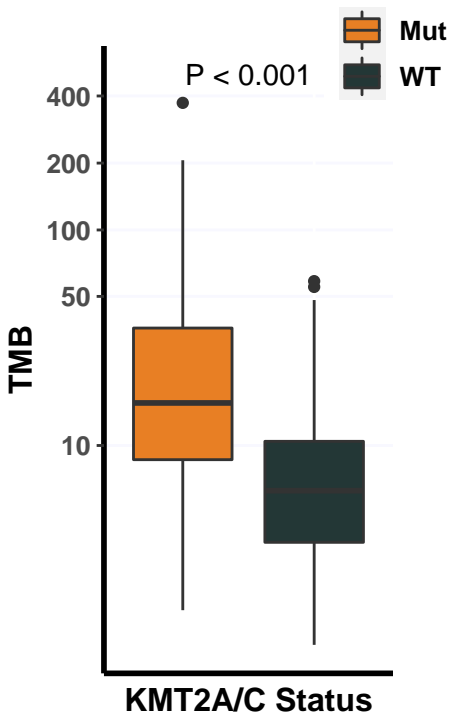

Supplement: Supplementary file 5 — Additional file 5: Figure S3. Boxplot comparing the tumor mutational burden (TMB) between KMT2A/C-Mut tumors and the KMT2A/C-Wt tumors in the primary ICI-treated cohort. [file 40364_2020_241_MOESM5_ESM.pdf]

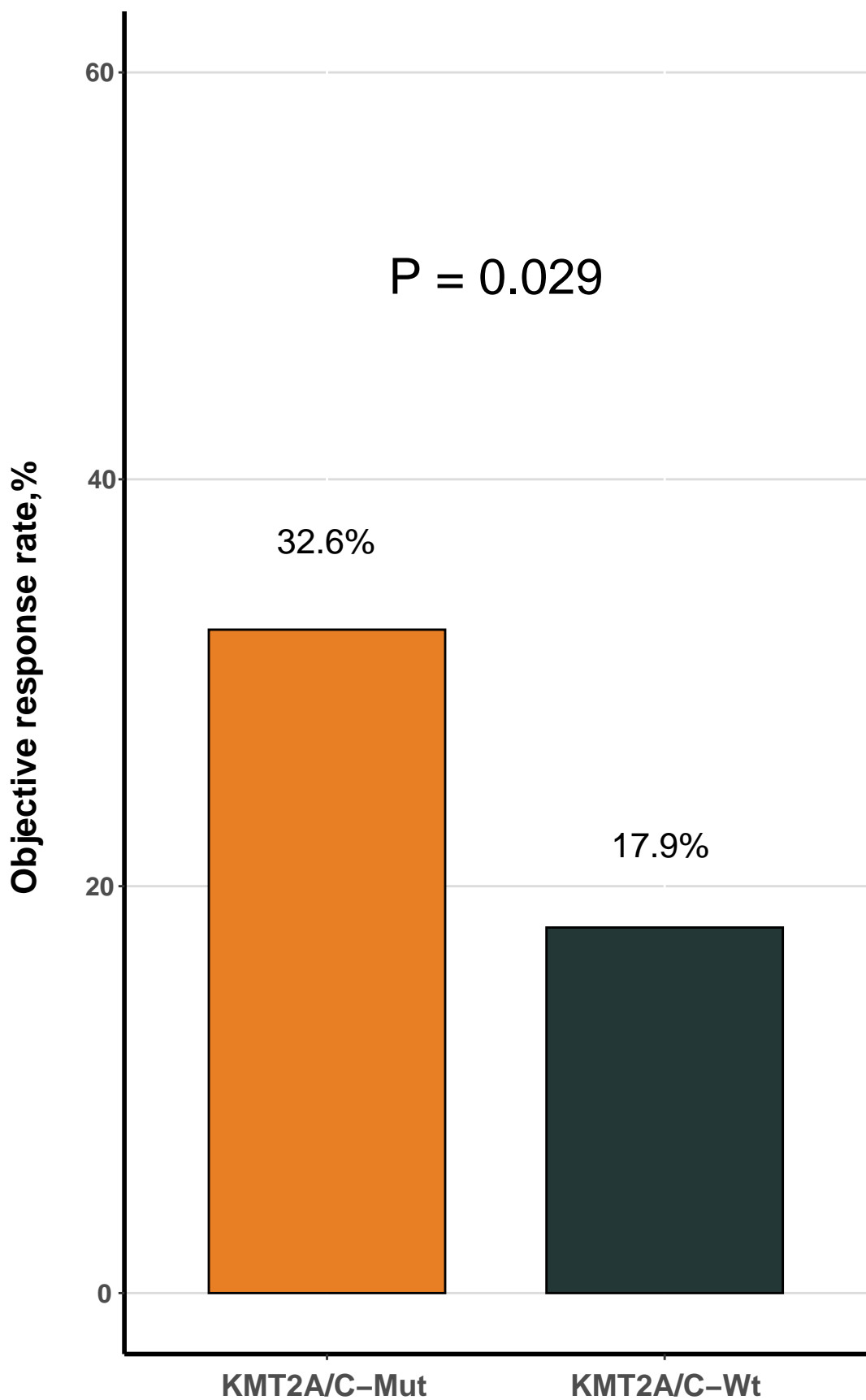

Supplement: Supplementary file 6 — Additional file 6: Figure S4. Barplot comparing the objective response rate (ORR) between KMT2A/C-Mut patients and the KMT2A/C-Wt patients in the TMB-low subgroup of the primary ICI-treated cohort. [file 40364_2020_241_MOESM6_ESM.pdf]
